# Peptide Targeted Gold Nanoplatform Carrying miR-145 Induces Antitumoral Effects in Ovarian Cancer Cells

**DOI:** 10.3390/pharmaceutics14050958

**Published:** 2022-04-28

**Authors:** Edison Salas-Huenuleo, Andrea Hernández, Lorena Lobos-González, Iva Polakovičová, Francisco Morales-Zavala, Eyleen Araya, Freddy Celis, Carmen Romero, Marcelo J. Kogan

**Affiliations:** 1 Departamento de Química Farmacológica y Toxicológica, Facultad de Ciencias Químicas y Farmacéuticas, Universidad de Chile, Santiago 8380000, Chile; es@aintech.cl (E.S.-H.); francisco.morales@umayor.cl (F.M.-Z.); 2 Advanced Center for Chronic Diseases (ACCDiS), Universidad de Chile & Pontificia Universidad Católica de Chile, Santiago 7820436, Chile; llobos@udd.cl (L.L.-G.); iva.polakovicova@email.cz (I.P.); 3 Advanced Integrated Technologies (AINTECH), Chorrillo Uno, Parcela 21, Lampa, Santiago 9380000, Chile; 4 Laboratory of Endocrinology and Reproduction Biology, Clinical Hospital, Universidad de Chile, Santiago 7820436, Chile; hernandez@hcuch.cl; 5 Centro de Medicina Regenerativa, Facultad de Medicina, Universidad Del Desarrollo, Santiago 7610658, Chile; 6 Department of Hematology-Oncology, School of Medicine, Pontificia Universidad Católica de Chile, Santiago 7820436, Chile; 7 Centro de Nanotecnología Aplicada, Facultad de Ciencias, Universidad Mayor, Temuco 4801043, Chile; 8 Departamento de Ciencias Quimicas, Facultad de Ciencias Exactas, Universidad Andres Bello, Santiago 8370146, Chile; eyleen.araya@unab.cl; 9 Laboratorio de Procesos Fotónicos y Electroquímicos, Facultad de Ciencias Naturales y Exactas, Universidad de Playa Ancha, Valparaíso 2360002, Chile; freddy.celis@upla.cl

**Keywords:** ovarian cancer, nanoplatform, microRNA

## Abstract

One of the recent attractive therapeutic approaches for cancer treatment is restoring downregulated microRNAs. They play an essential muti-regulatory role in cellular processes such as proliferation, differentiation, survival, apoptosis, cell cycle, angiogenesis, and metastasis, among others. In this study, a gold nanoplatform (GNPF) carrying miR-145, a downregulated microRNA in many cancer types, including epithelial ovarian cancer, was designed and synthesized. For targeting purposes, the GNPF was functionalized with the FSH33 peptide, which provided selectivity for ovarian cancer, and loaded with the miR-145 to obtain the nanosystem GNPF-miR-145. The GNPF-mir-145 was selectively incorporated in A2780 and SKOV3 cells and significantly inhibited cell viability and migration and exhibited proliferative and anchor-independent growth capacities. Moreover, it diminished VEGF release and reduced the spheroid size of ovarian cancer through the damage of cell membranes, thus decreasing cell viability and possibly activating apoptosis. These results provide important advances in developing miR-based therapies using nanoparticles as selective vectors and provide approaches for in vivo evaluation.

## 1. Introduction

Nanotechnology has emerged as a feasible alternative to develop safe and effective therapeutic agents according to nanoparticle properties. The capacity to modulate a nanoparticle’s size, morphology, and surface chemistry allows the creation of flexible nanosystems, with almost inexhaustible conformational possibilities [1]. Moreover, the incorporation of active agents in a nanostructure permits its protection against degradation by light, oxidation, or enzymatic attack. Furthermore, the accumulation of several active molecules inside a nanostructure or in its surroundings, confines them to a small volume, allowing more concentrated doses of the active agent to the target organ or cell type, thus promoting a better biological response [2].

Among the wide range of nanosystems investigated in nanomedicine, gold nanoparticles (AuNPs) have been extensively studied due to their inherent optical properties, low toxicity, and ability to carry higher quantities of molecules on their structures. Their surface chemistry is easily modifiable, and molecules can be loaded via different mechanisms, such as covalent or ionic bonds [3,4,5]. The protection offered has been proven even for very unstable molecules in biological media, such as short RNA strands. Moreover, AuNPs can be multifunctionalized at their surface, affording the possibility to conjugate other kinds of molecules to obtain differentiated properties in only one nanosystem. For example, targeted selectivity has used ligand molecules for specific cell receptors, thus avoiding off-target side effects, and reporter molecules such as luminescent or fluorescent tags allow monitoring of their organ or tissue accumulations. Thus, AuNPs offer the possibility to develop theranostic nanosystems with the ability to detect and treat pathologies at the same time [6,7,8].

A plethora of gold nanosystem compositions have been developed to carry many types of bioactive or therapeutic molecules for cancer disease [9]. The better performance of antineoplastic drugs, such as platin-derived alkylating agents or antibodies, for example, has been evidenced under nanosystem administration [10]. Recently, the use of microRNAs (miR), biogenic molecules with an essential role in gene expression regulation at the posttranscriptional level, has been effective in suppressing the messengers that are aberrantly upregulated in cancer phenotypes. The incorporation of various miR in gold nanoplatforms has been reported so far, but none of them has been structured to obtain an organ- or tissue-selective nanosystem [11].

Thus, we designed a gold nanoplatform (GNPF) that specifically targets ovarian cancer cells, based on gold nanospheres (GNS) carrying miR-145 as the active molecule, conjugated with 5 kDa polyethylene glycol (HS-PEG-NH_2_) and the FSH33 peptide as a ligand for the FSH receptor specifically expressed in ovarian cells [12]. The novelty of this work as new potential adjuvant therapy for ovarian cancer using gold nanoparticles is: (a) to protect miR-145 from ribonucleases and long noncoding RNAs (lncRNAs) that can act as sponges of miR-145, preventing its effects and (b) the incorporation of a FSH fragment in the architecture of the AuNP nanosystem to ensure that GNPF-miR-145 reaches only ovarian cancer cells [12]. We characterized the nanoplatform, evaluated its efficacy against ovarian cancer cells in a 2D model without using transfection agents, and validated the results in 3D models as the initial target. We studied the incorporation of the nanosystem into the cells by quantifying gold content, miR molecules, and the main cellular effects, such as proliferation, migration, clonogenicity, and VEGF delivery. This nanoplatform protected the miR molecule, achieving a cell-selective biological antitumoral response in an in vivo-like cell context, such as a 3D scenario. Thus, we present it as a viable platform to be used in the next step of preclinical investigations.

## 2. Materials and Methods

### 2.1. Reagents

If not specified, reagents were purchased from Sigma-Aldrich (St. Louis, MI, USA).

### 2.2. Gold Nanosystem Preparation

GNS were obtained by the sodium citrate reduction method. Briefly, 100 mL of a solution of 1 mM HAuCl_4_ was refluxed for 15 min at 185 °C, then 10 mL of a solution of 38.8 mM sodium citrate was quickly added. The reflux was continued for 30 min until a deep red suspension was obtained. The resultant suspension was filtered through a 0.22 µm syringe filter to remove any precipitate. To prepare GNS coated with 5 kDa polyethylene glycol with sulfhydryl and amine ends (PEG), 20 mL of 5 nM GNS was prepared, and 4 mg of PEG in 1 mL of Milli-Q (MQ) water was added dropwise under magnetic stirring and allowed to react for 5 h. To eliminate the non-reacting PEG, the suspension was centrifuged for 30 min at 16,000× *g*, the supernatant was discarded, and the pellet was resuspended in MQ water. This procedure was performed at least three times to eliminate the PEG excess, obtaining the GNS-PEG construct.

To functionalize the GNS-PEG with the FSH33 peptide (YTRDLVYKDPARPKIQKTCTF) and obtain the GNPF (GNS-PEG-FSH33), a crosslinking reaction was performed. First, 0.5 mg of FSH33 was solubilized in 500 µL of 4-morpholineethanolsulfonic acid buffer (MES) at pH 5, then a solution of 500 µL of 10 mg of 1-ethyl-3-(3-dymethylaminopropyl (EDC) and 25 mg of N-hydroxysuccinimide (NHS) in MES was added. The mixture was sonicated in an ultrasound bath for 15 min and agitated for another 15 min. Then, the activated FSH33 peptide was added dropwise to 20 mL of GNS-PEG 5 nM under agitation, incubating it for 3 h. The resultant suspension was centrifuged for 30 min at 16,000× *g*, repeating this procedure at least three times to assure the elimination of peptide excess, obtaining the GNPF.

To load the miR145 or scrambled miR on the GNPF surface, 0.5 µL of miR-145 25 µM was mixed with 1 mL of GNPF 1 nM and incubated for 15 min under agitation. Once the GNPF-miR-145 constructs were obtained, they were used immediately for the characterization procedures and in vitro assays.

### 2.3. Molecular Absorption Spectrophotometry

UV-Vis spectra of the nanosystems were obtained with a Lambda UV25 Perkin Elmer spectrophotometer, measuring the absorbance in the range of 300–800 nm using quartz vessels. For naked GNS, 1.2 mM sodium citrate was used, and GNS-PEG-NH_2_ and the GNPF were diluted in Milli-Q water.

### 2.4. Raman Spectroscopy

The Raman spectrum of FSH33 and SERS spectra of GNS-PEG and GNPF were recorded using an Alpha 300 SNOM/Raman microscope equipped with a 785 nm laser line. The spectra were recorded using the 20× objective in the range 100–1750 cm^−1^, and the number of acquisitions was set at 10, with 10 s of integration time. Samples were placed on a thin sheet of gold to avoid any intrinsic fluorescence that might be emitted by the amino acids Tyr and Phe. The gold surface was prepared by sputtering deposition of the metal on a glass substrate under Argon plasma. This methodology avoided any interference caused by fluorescence emission in the respective Raman spectrum.

### 2.5. Dynamic Light Scattering (DLS)

DLS measurements were performed using the Nano ZS instrument (Malvern, UK) operating with a fixed backscatter detector angle of 173° with the cell holder maintained at 25 °C. Data were collected, allowing the instrument to automatically optimize the measurement parameters (attenuator and number of runs) using a disposable polycarbonate capillary cell (DTS 1061, Malvern, UK). The hydrodynamic diameter of each sample was the result of at least three acquisition steps with an average of 12–16 consecutive runs for each step.

### 2.6. Zeta Potential (ƺP)

The ƺP was determined using the Nano ZS instrument (Malvern, UK) with the same conditions as DLS determination. Since these measurements were performed in an aqueous solution, the Smoluchowski approximation was used to calculate the ƺP based on the measured electrophoretic mobility. The determined ƺP was the result of at least three acquisition steps, with an average of 100 measurements for each step.

### 2.7. Estimation of miR Coated on Nanoparticles

To estimate the number of miR-145 molecules on the surface of the GNPF, increasing concentrations of miR-145 labeled with the Cy5 fluorophore (1.56, 3.13, 6.25, 12.5, 20, and 50 nM) were measured in triplicate. For measurements and calibration curves, the 650 and 670 nm wavelengths for excitation and emission were used. Then, after coating the GNPF with miR-145, the dispersion was centrifuged at 1500× *g* for 1 h. The supernatant was recovered, and the fluorescence was measured in a Biotek multiscan^®^ plate reader. The concentration of miR-145 was determined by subtraction against the concentration used.

### 2.8. Scanning Transmission Electronic Microscopy (STEM)

Ten microliters of each nanosystem were loaded onto formvar-carbon copper grids of 300 mesh (Electron Microscopy Science, Hatfield, UK) and left to dry for 24 h at room temperature. Samples were visualized in an FEI INSPECT F50 high-resolution scanning transmission electron microscope. For determination of size, 200 nanoparticles were counted.

### 2.9. Cell Viability

Cell viability was evaluated in HeLa, ATCC A2780 and SKOV3 cell lines from a cell culture close to 70% confluence. A total of 2500 cells were seeded per well in 96-well plates the day prior to the treatments. On the second day, cells were incubated with increasing concentrations of each nanosystem. Exposure to the treatments lasted 24 h, and the CellTiter 96^®^ AQueous colorimetric assay (Promega, Madison, WI, USA) was used to determine the absorption at 490 nm, in a Synergy H2 model Biotek plate reader (Santa Clara, CA, USA). The data were obtained at least in triplicate, with *n* = 12.

### 2.10. Nanoparticle Cell Incorporation

A2780 cells (1,000,000) were seeded per well in 6-well plates. To facilitate the adherence of the cells, the following treatments were applied after 4 h: DMEM/SFB medium 10% (control), miR-145 25 nM, GNS-PEG 1 nM, GNPF-miR-145 1 nM, chemotherapy (8 µM paclitaxel + 50 µM carboplatin) and GNPF-miR-145 + chemotherapy (1 nM GNPF-miR-145 + 8 µM paclitaxel + 50 µM carboplatin). The cells were incubated for 0, 3, and 24 h. The adhered cells were washed 3 times with PBS, and then 500 µL of HNO_3_ was added directly to the well, the cells were resuspended, and then allowed to act for 15 min. Subsequently, the volume was transferred into 1.5 mL conical tubes and incubated for 30 min at 70 °C in a water bath. Finally, the tubes were incubated for 48 h before the measurements. For the quantification of gold, a calibration curve was prepared with gold-standard solutions (Merk) of concentrations 0.001, 0.005, 0.01, 0.1, 1, and 10 ppm in HNO_3_ 2% *w/v*. We reached a correlation coefficient R^2^ > 0.99999 and a relative standard error <2%. The digested samples were diluted by factors of 10, 100, and 1000 in 2% HNO_3_, and their concentration was determined by atomic emission spectroscopy with an Agilent^®^ 4100 MP-AES (Santa Clara, CA, USA) microwave plasma atomic emission spectrometer, using the mainline for gold at 267,595 nm. Measurements were made in triplicate with *n* = 3 for each point.

### 2.11. miR Quantification

A2780 cells (500,000) were seeded per well in 6-well plates. To facilitate cell adherence to the bottom of the plate, the following treatments were applied after 4 h: DMEM/SFB 10% medium (control), miR-145 25 nM, GNPF-miR-145 1 nM, chemotherapy (8 µM paclitaxel + carboplatin 50 µM) and GNPF-miR-145 + chemotherapy (1 nM GNPF-miR-145 + 8 µM paclitaxel + 50 µM carboplatin). The cells were incubated for 0, 3, and 24 h. At each end time, the supernatant was discarded, and the cells were washed with PBS. Subsequently, the cells were treated with 500 µL of TRIzol^®^ reagent, homogenized, and stored at −80 °C. For the extraction of total RNAs, the miRNeasy mini kit (Qiagen, Venlo, The Netherlands) was used. To perform the reverse transcription of the miRNAs copied to cDNA, the miScript II RT kit (Qiagen) was used according to the supplier’s protocol. SuperScript II reverse transcriptase (Invitrogen, Waltham, MA, USA)), random primers (Promega, Madison, WI, USA), and dNTPs in concentration 10 Mm (Promega) were used. For the detection and quantification of miR145, the miScript SYBR Green qPCR kit (Qiagen) was used in a StepOne Real-Time PCR System (Applied Biosystems, Waltham, MA, USA) according to the supplier’s instructions. Endogenous RNA RNU6 was used as a control and normalizer, and sterile water was used as a negative control.

### 2.12. Proliferation Assay

Cell proliferation was evaluated in the A2780 and SKOV3 epithelial ovarian cancer cell lines since the initial cell culture of each cell line was close to 70% confluency. A total of 2500 cells were seeded per well in 96-well plates the day prior to the treatments. On the second day, the cells were incubated with the following treatments: GNPF, GNPF-miR-145, GNPF-SC-miR, miR-145, chemotherapy (8 µM paclitaxel + 50 µM carboplatin), and GNPF-miR-145 + chemotherapy. DMEM/SFB 10% and SDS 10% were used as controls. The cells were treated for 24, 48, and 72 h, and the CellTiter96^®^ AQueous colorimetric assay was performed, determining the absorption at 490 nm. The data were obtained in triplicate, with *n* = 4.

### 2.13. Wound Healing Assay

Cell migration assays were performed for the SKOV3 cell line using the wound healing method. With this in mind, from an initial cell culture of 70% confluence, 50,000 cells were seeded per well in 96-well plates with DMEM/10% SFB medium for 24 h. On the second day, a 100% confluence was verified, the culture medium was removed, washed with sterile PBS, exchanged for DMEM/1% SFB medium, and the cells were treated with: GNPF, miR-145, GNPF-miR-145, chemotherapy, and GNPF-miR-145 + chemotherapy, incubating for 24 h. On the third day, changes in the cell monolayer were verified by visual inspection, and a vertical wound was made with a sterile 200 µL tip in each well, using the lid of the 96-well plate as a guide. The culture medium was then removed, the cells were washed with sterile PBS to remove cells and debris that were released after wound formation, and the medium was renewed with DMEM/10% SFB. Images of the wound formed in each well were captured at 0, 8, and 21 h on an Olympus U-CMAD3 inverted light microscope, using a Nikon DS-F12 camera. Subsequently, the images were analyzed using the ImageJ software, measuring the distance between the borders of both sides of the wound at each time, for each well, taking as reference the same image at time 0 of each well as 100% opening (0% recovery). Data were obtained in triplicate with *n* = 4.

### 2.14. VEGF Release

VEGF release was evaluated with an ELISA immunoassay. The culture medium of A2780 cells was incubated for 0, 3, and 24 h with miR-145 treatments, GNPF-miR-145, chemotherapy and GNPF- miR-145 + chemotherapy, and centrifuged at 4000× *g* for 5 min before the test. VEGF detection was performed with the Quantikine Human VEGF Immunoassay kit (R&D systems DVEOO, Minneapolis, MN, USA). For this, 50 µL of RD1W diluent was added to each well, followed by 200 µL of supernatant. The plate was covered with adhesive strips and incubated for 2 h at room temperature. Then, the volume of each well was removed and washed with 400 µL of buffer, aspirating as a final step. A 200 µL amount of Human VEGF conjugate was added to each well and incubated for 2 h at room temperature, washing as in the previous step. Subsequently, 200 µL of the substrate solution was added to each well, incubating for 20 min at room temperature, protecting from light. Then, 50 µL of stop solution was added to each well. The absorbance determinations were carried out in a 96-well plate reader (Biotek EL800, Santa Clara, CA, USA) at 450 nm. The data obtained were the result of triplicate samples for each time.

### 2.15. Endothelial Cell (EA.hy926) Proliferation with Conditioned Medium from EOC Cells

Cell proliferation of the EA.hy926 human endothelial line was evaluated with wound assay after being exposed to conditioned medium of A2780 ovarian cancer cells. For this, A2780 cells were seeded in 24-well plates and treated for 24 h with GNPF, miR-145, GNPF-miR-145, chemotherapy, and GNPF-miR-145 + chemotherapy. Then, the conditioned medium was collected and centrifuged at 4000× *g* for 5 min to remove cells and debris. This medium was used to treat EA.hy926 cells for 48 h, which were seeded in 96-well plates with a density of 2500 cells per well the day before the treatments. Once the exposure time had elapsed, the CellTiter 96^®^ AQueous colorimetric assay was performed, determining the absorption at 490 nm. The data were obtained in triplicate, with *n* = 4.

### 2.16. Clonogenic Assay

The clonogenic capacity of the A2780 cell line was assessed. For this, a growth curve of tumoroids was first calculated with increasing cell concentrations, determining the optimal number of cells to be seeded to obtain quantifiable tumoroids. A total of 7000 cells were seeded per well in 6-well plates in soft agar, in triplicate, with the following treatments: DMEM/SFB 10% medium (control), GNPF, GNPF-SC-miR 1 nM, miR-145 25 nM, GNPF-miR-145 1 nM, chemotherapy (8 µM paclitaxel + 50 µM carboplatin) and GNPF-miR-145 + chemotherapy (1 nM GNPF-miR-145 + 8 µM paclitaxel + 50 µM carboplatin). Cells were incubated for 14 days, and clones with diameters of 100 µm formed under each treatment were counted by light microscopy.

### 2.17. Cancer Tumoroid Model

Tumor spheroids from SKOV3 lines were formed by the liquid overlay technique method. Ninety-six-well plates were coated with 50 µL of 1.5% agarose in DMEM. From cells with 70% confluence, 5000 cells were seeded per well (after studying growth curves with different numbers of initial cells, to obtain spheroids of 500 µm in diameter in a period of 4–6 days). Subsequently, the plate was shaken at 120 rpm for 1.5 h on an orbital shaker at 37 °C. The formation and growth of the spheroids were visualized in an Olympus U-CMAD3 inverted optical microscope, using a Nikon DS- F12 camera, acquiring images of each spheroid at times 0, 3, 6, 7, and 11 days. On the sixth day, the spheroids were exposed to the different treatments: 10% DMEM/SFB medium (control), 25 nM miR-145, 1 nM GNPF-miR-145, chemotherapy (8 µM paclitaxel + 50 µM carboplatin), and GNPF-miR-145 + chemotherapy (1 nM GNPF-miR-145 + 8 µM paclitaxel + 50 µM carboplatin). The morphological effects were evaluated by light microscopy at 0, 1, 2, 3, and 5 days of treatment.

Image analysis was performed using the ImageJ software, determining the area and diameter of each spheroid at each time. On the fifth day, intact spheroids were assessed for cell viability with the MTS, LDH, and Annexin V/propidium iodide assays.

## 3. Results

### 3.1. Nanoplatform Obtention and Characterization

**Figure 1:**
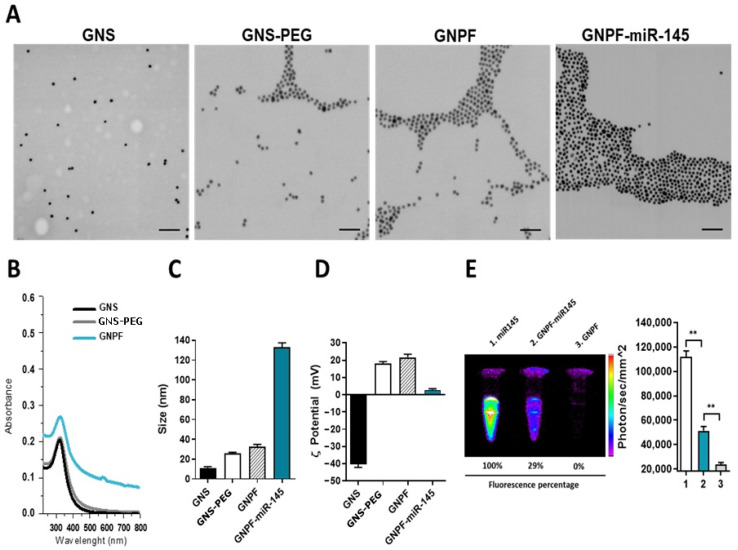
**Physicochemical characterization of the gold nanosystems.** (**A**) Representative TEM micrographs for each step of the functionalization process. The spherical morphology of gold nanoparticles with an average size of 12 nm can be observed. The scale bar represents 100 nm. (**B**) Molecular absorption spectrophotometry of the nanosystems, showing the absorption bands that correlated to the plasmon resonance effect in the gold nanospheres. (**C**) Characterization of the hydrodynamic diameter in each step of the functionalization. It is possible to appreciate the increment in the size as other molecules coated on the nanoparticle surface. (**D**) ƺ potential determinations evaluated in each functionalization. Changes in magnitude occurred when molecules with opposite charge were conjugated. (**E**) Fluorescence image (**left**) and its quantification (**right**) of the GNPF functionalized with the miR145 coupled to the Cy5 fluorophore. It can be visualized that the miR-145 fluorescence is quenched when it is present on the nanoparticle surface. 1 = miR145; 2 = GNPF-miR145; 3 = GNPF; ** = *p <* 0.001.

The GNPF was physicochemically characterized by its size, hydrodynamic diameter, morphology, surface charge, plasmonic absorption band, vibrational Raman/SERS profile and the respective changes in these parameters, according to the successive chemical modifications on the nanoparticle surface. In Figure 1A (left), the GNS with a size of 12 ± 2 nm is shown. All of the modifications for the nanoparticles showed the characteristic absorption spectrum shape, corresponding to GNS [13], with a defined maximum absorption band close to 520 nm, which correlated with sizes from 10 to 30 nm (Figure 1B). Unconjugated GNS exhibited an average hydrodynamic diameter of 12 nm, with homogeneous distribution and negative surface charge of −40 mV, similar to that reported previously for the citrate reduction method [14]. When conjugating GNS with HS-PEG-NH_2_ (PEG) to obtain GNS-PEG, we observed that the hydrodynamic diameter increased to 25 nm, and the charge was inverted to positive, at 18 mV. This indicated the incorporation of the PEG molecules (with amine positively charged) on the nanoparticle surface, giving a molecular layer 6–7 nm thick, consistent with PEGylations with PEGs of molecular weight 5 kDa on GNS of 13 nm [15] and a positive ƺ potential (Figure 1C,D, supporting information in Figure S1). In order to corroborate that PEG was bonded onto the surface of GNS, the corresponding SERS spectrum (Figure S2A) of the GNS-PEG system was analyzed and assigned. The strong band observed around 270 cm^−1^ (Figure S2B) and ascribed to the Au-S stretching mode demonstrated that the conjugation between GNS and PEG occurred on the nanostructured surface and through the sulfur atom. Coupled vibrational modes attributed to the -CH_2_, C-OH and C-C moieties appeared at 800, 867, 1080 and 1117 cm^−1^. Finally, the signal observed at 1048 cm^−1^ was assigned to the C-N stretching vibration and was associated with the amine terminal of PEG [16,17]. When the GNS-PEG was functionalized with the FSH33 peptide to obtain the GNPF, we observed that the hydrodynamic diameter increased to 35 nm, which correlated with the incorporation of new molecular species maintaining a positive ƺ due to the positive charge of the peptide at pH = 7.4 (isoelectric point 9.56). In the corresponding SERS spectrum (Figure S2A) of the new GNPF nanosystem, at least 6 new bands associated with the FSH33 peptide could be observed and assigned. The signal ascribed to the Au-S stretching mode and related to the functionalization with PEG showed a downshift of 10 cm^−1^ (Figure S2B) in the spectrum due to the incorporation of the FSH33 peptide. Some of new bands, as in case of 1135 and 1157 cm^−1^, were attributed to the C-N stretching mode of the new amide functional group. Similarly, two other new bands, appearing at 815 and 828 cm^−1^, were also ascribed to the C-N stretching mode of the amide group. It is relevant to mention that these new bands were not observed in the Raman spectrum of the peptide (Figure S2A), being evidence of the formation of the new nanosystem. In addition, new SERS bands associated with the amino acids Phe (964 and 1003 cm^−1^) and Cys (652 cm^−1^) were observed [17,18]. Therefore, the analysis of the SERS spectrum of GNPF demonstrated the functionalization of GNS-PEG with the FSH33 peptide.

As a final step, the inclusion of the miR-145 molecules into the nanosystem forming GNPF-miR-145 showed that the size increased to 134 nm (Figure 1C) with a diminution of the absolute ƺ potential value due to the negative charge of the miR-145. The increase in particle size and surface charge were consistent with the incorporation of molecular species. The fluorescence quenching emitted by the CY5 label in the RNA molecule was due to the interaction with the plasmon nanoparticle’s surface [19] (Figure 1E).

### 3.2. Gold Nanoplatforms Do Not Affect Cell Viability and Selectively Interact with Ovarian Cancer Cells

**Figure 2:**
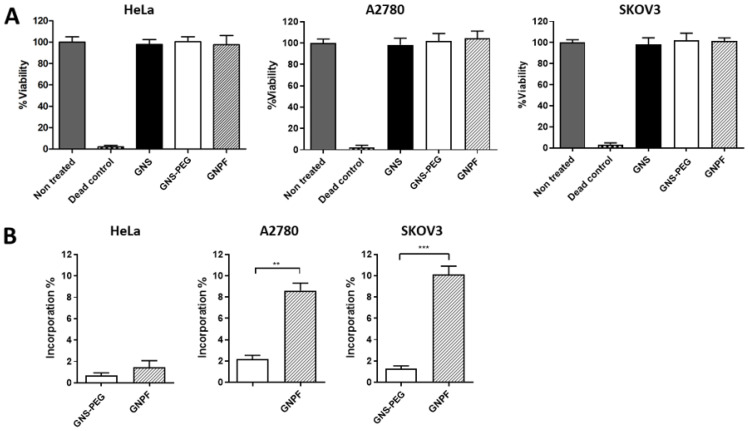
**Cell viability and cell interaction of the conjugated nanosystems, previous to the functionalization with miR145.** (**A**) Viability MTS assay in HeLa, A2780, and SKOV3 cell lines after 24 h incubation with 3.2 nM of GNS, GNS-PEG, and GNPF. *n* = 10. (**B**) Evaluation of the GNS-PEG and GNPF presence in HeLa, A2780, and SKOV3 cell lines after 24 of exposure. *n* = 5. For all graphics, error bars represent the standard deviation; ** = *p* < 0.01; *** = *p* < 0.001.

The nanosystems were incubated with HeLa (uterine), A2780 (ovarian), and SKOV3 (ovarian) cultured cell lines to assess the viability and cell interaction effects. The results showed that neither the incomplete nanosystems (GNS or GNS-PEG) nor GNPF provoked a deleterious effect on viability in the three mentioned cell lines (Figure 2A). In addition, the presence of GNPF inside the cells was significantly higher in the ovarian cancer cell lines (that overexpress the FSH receptor [12]) compared with the uterine cell line, and it was superior to GNS-PEG, having a positive charge (Figure 2B). The results indicate that GNPF does not evoke a negative viability response and can reach and selectively interact with ovarian cancer cells, probably due to the conjugation with the FSH33 ligand, whose receptor is only present in ovarian cells and upregulated in ovarian cancer cells.

### 3.3. GNPF-miR-145 Can Reach the Cells and Deliver miR-145

**Figure 3:**
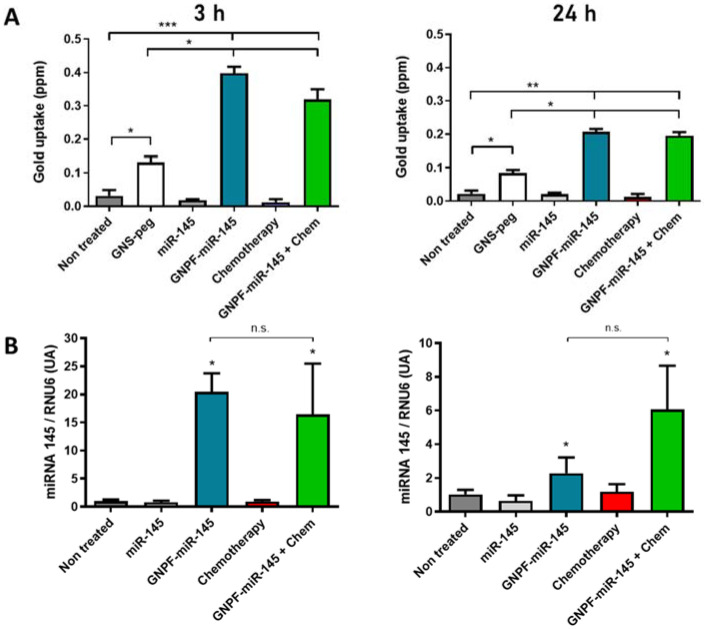
**Quantification of cellular incorporation of GNPF-miR-145**. (**A**) Gold quantification in A2780 cells at 3 and 24 h. The data were obtained in triplicate; *n* = 3; error bars = standard deviation. (**B**) miR-145 quantification. Normalized miR-145 levels in cells treated with free miR-145, GNPF-miR-145, chemotherapy and GNPF-miR-145 + chemotherapy, evaluated at 3 and 24 h (left and right graphs, respectively). * = *p* < 0.05, ** = *p* < 0.01 and *** = *p* < 0.001 with respect to the miR-145 control; n.s. = statistically not significant; *n* = 4; error bars = standard deviation.

To evaluate whether the GNPF-miR-145 reached the ovarian cancer cells and delivered miR-145, gold and miR-145 were quantified after the incubation on A2780 cells for 0, 3, and 24 h. No transfection agent was used in order to imitate the in vivo context and be able to evidence internalization only based on the GNPF-miR-145 physicochemical interactions. The results show that cells treated with GNS-PEG, GNPF-miR-145, and GNPF-miR-145 + chemotherapy (8 µM paclitaxel + carboplatin 50 µM) had significantly higher gold concentrations than untreated control and free miR-145, indicating that gold nanosystems were internalized in the treated cells. Thus, after 3 h exposure, when comparing the GNPF-miR-145 incorporation with the control nanosystem GNS-PEG, it was evidenced that the GNPF-miR-145 was uptaked three times more (Figure 3A). The GNS-PEG system had a primary amine at its end, with a positive charge of 18 mV (Figure 1D), which favored entry into the cells by electrostatic interaction with the negative charges of the phosphate groups of the phospholipids at the cell membrane. While GNPF-miR-145 had a net charge close to neutrality (Figure 1D), compared to the GNS-PEG system, its charge did not favor cell entry through electrostatic interaction; however, the higher concentration found in cells would be due to the FSH33 ligand at its end. This ligand recognizes the FSH receptor overexpressed in ovarian cancer cells, favoring nanoparticle–cell interaction and facilitating entry into the cells, possibly by receptor-mediated endocytosis and its consequent selective internalization, as previously reported [20]. The miR-145 content was quantified by qRT-PCR. The results indicate that miR-145 levels at time 0 were not significantly different between all treatments. Nevertheless, after 3 h exposure, the miR-145 levels in cells treated with GNPF-miR-145 or GNPF-miR-145 + chemotherapy were up to 20 times higher than in cells treated with free miR-145 or the untreated control. Both gold and miR content quantification indicated that GNPF-miR-145 entered the cells and effectively delivered the miR-145 cargo (Figure 3B). There were no significant differences between the GNPF-miR-145 and GNPF-miR-145 + chemotherapy treatments regarding miR-145 levels (Figure 3B). This indicates that chemotherapy administered together with GNPF-miR-145 did not interfere with the uptake mechanism, thus enhancing or adding the cellular effects.

After 24 h incubation, a decrease in both gold and miR-145 quantities was observed in cells treated with GNPF-miR-145 compared to 3 h incubation. 

### 3.4. Cell Viability and Antiproliferative Effects

**Figure 4:**
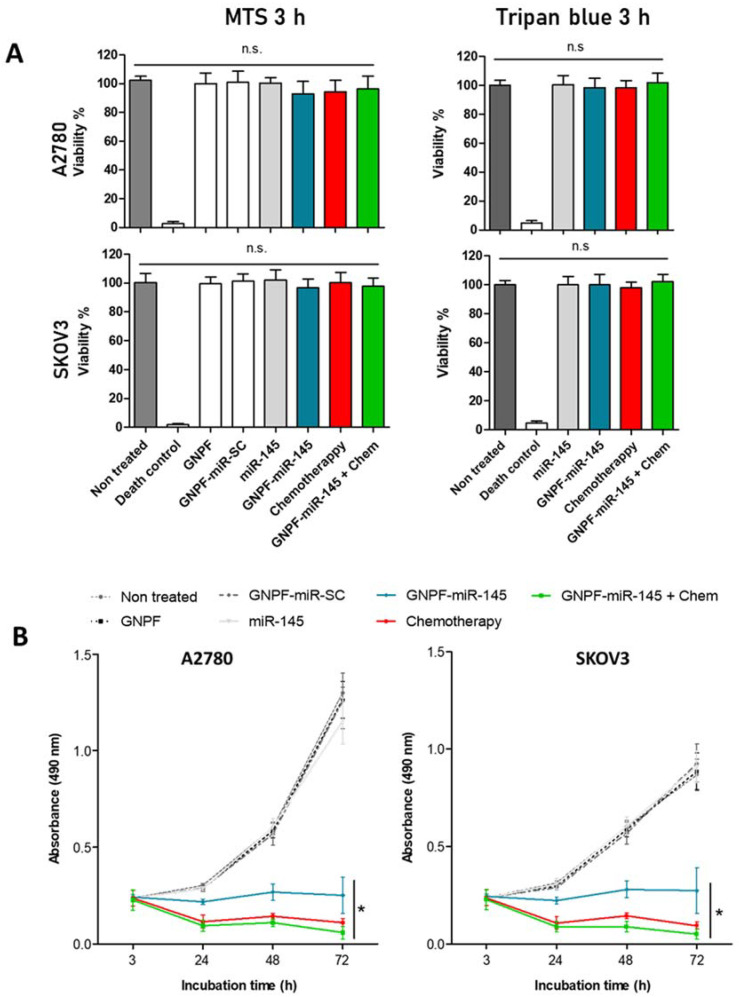
**Cell viability and proliferation after incubation with the final nanosystems.** (**A**) MTS viability and trypan blue exclusion assays in A2780 and SKOV3 cell lines after 3 h incubation with 1 nM of GNPF, GNPF-SC-miR, miR-145, GNPF-miR-145, chemotherapy, and GNPF-miR-145 + chemotherapy. *n* = 3, n.s. = not significant. (**B**) Proliferation assay using MTS reaction at 3, 24, 48, and 72 h exposure to the nanosystems. For all graphics, error bars represent the standard deviation, * = *p* < 0.05.

To evaluate whether the nanosystems provoked toxic effects in cells when the major accumulations were found, cell viability assays were performed in A2780 and SKOV3 cell lines. The cells were exposed for 3 h to each nanoparticle system, with and without the presence of miR-145 or scrambled miR. There were no significant differences concerning the control; therefore, the nanosystem did not affect cell viability in the lines evaluated (Figure 4A). The GNPF-miR-145 effects on cell proliferation were evaluated after 24, 48, and 72 h of incubation with the nanosystems. At these times, the effect could be reflected in proliferation, since several cycles of cell division had already occurred. When analyzing the effects over time, a significant decrease in proliferation was observed in cells treated with GNPF-miR-145, chemotherapy, and GNPF-miR-145 + chemotherapy, both at 48 and 72 h (Figure 4B). First, an initial decrease in cell viability was observed. This suggests an initial cytotoxic effect caused by GNPF-miR-145, chemotherapy, and GNPF-miR-145 + chemotherapy, with an 8, 58, and 67% reduction in viability for the A2780 line and a reduction of 12, 54, and 62% for the SKOV3 line. At the same time, GNPF-miR-145 reduced cell proliferation by 23% in both cell lines in comparison with the free miR-145, GNPF, and GNPF with a scrambled miR (GNPF-SC-miR). The chemotherapy treatment showed a decrease of 63%; in turn, exposure of cells to both treatments reduced proliferation by up to 70%. After 48 h, a clear effect on cell proliferation was observed. GNPF-miR-145 provoked decreases of 48 and 55% in the proliferation of A2780 and SKOV3 cell cultures, respectively. Chemotherapy and GNPF-miR-145 + chemotherapy treatments decreased proliferation by 76% and 83%, respectively in both cell lines, showing significant differences comparing the control. Likewise, when evaluating the effect at 72 h, GNPF-miR-145 reduced proliferation by up to 76 and 69%, chemotherapy by up to 92 and 89%, and both treatments by up to 96 and 94%, respectively for A2780 and SKOV3 (Figure 4B).

### 3.5. Nanoplatform Carrying miR-145 Inhibits Cell Migration

**Figure 5:**
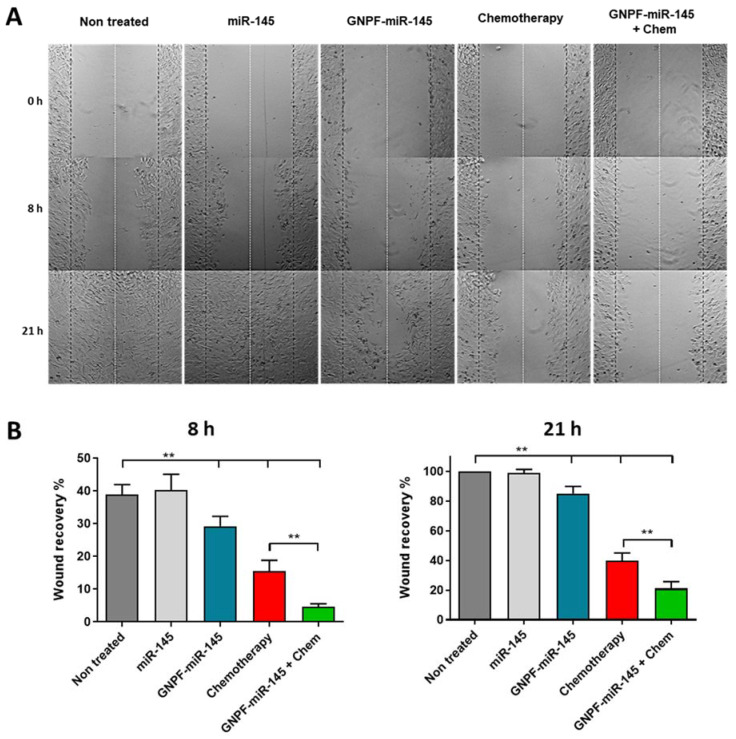
**Scratch wound healing assay.** (**A**) Time-lapse optical micrographs at 0, 8, and 21 h of SKOV3 cell line in the healing process after 24 h of incubation with the treatments. (**B**) Graphics of the wound recovery at 8 and 21 h. The experiment was performed in triplicate, *n* = 6. For all graphics, error bars represent the standard deviation, ** = *p* < 0.01.

The ability of SKOV3 ovarian cancer cells to migrate after 24 h exposure to GNPF-miR-145 was evaluated through the wound healing assay. The results indicated that free miR-145 did not exert effects on cell migration, behaving the same as the untreated control. Regarding GNPF-miR-145, a migration inhibition effect was observed starting at 8 h of wound healing time, with 10% less recovery than controls, noting that at 21 h the wound did not close completely, reaching around 85% recovery. Chemotherapy resulted in a recovery rate of 40% at 21 h, while the effect of exposure to both treatments resulted in a recovery rate of only 21% (Figure 5).

### 3.6. GNPF-miR-145 Inhibits VEGF Release from A2780 Cells

Ovarian cancer cells constantly secrete vascular proliferation factors such as VEGF at the interstice, thus intensely promoting angiogenesis and forming highly irrigated big-sized tumors. Therefore, VEGF translation inhibition has a direct effect on endothelial cell proliferation and de novo blood vessel formation. It has been widely reported that miR-145 is a direct inhibitor of VEGF messenger, decreasing its intracellular and secreted levels [21].

**Figure 6:**
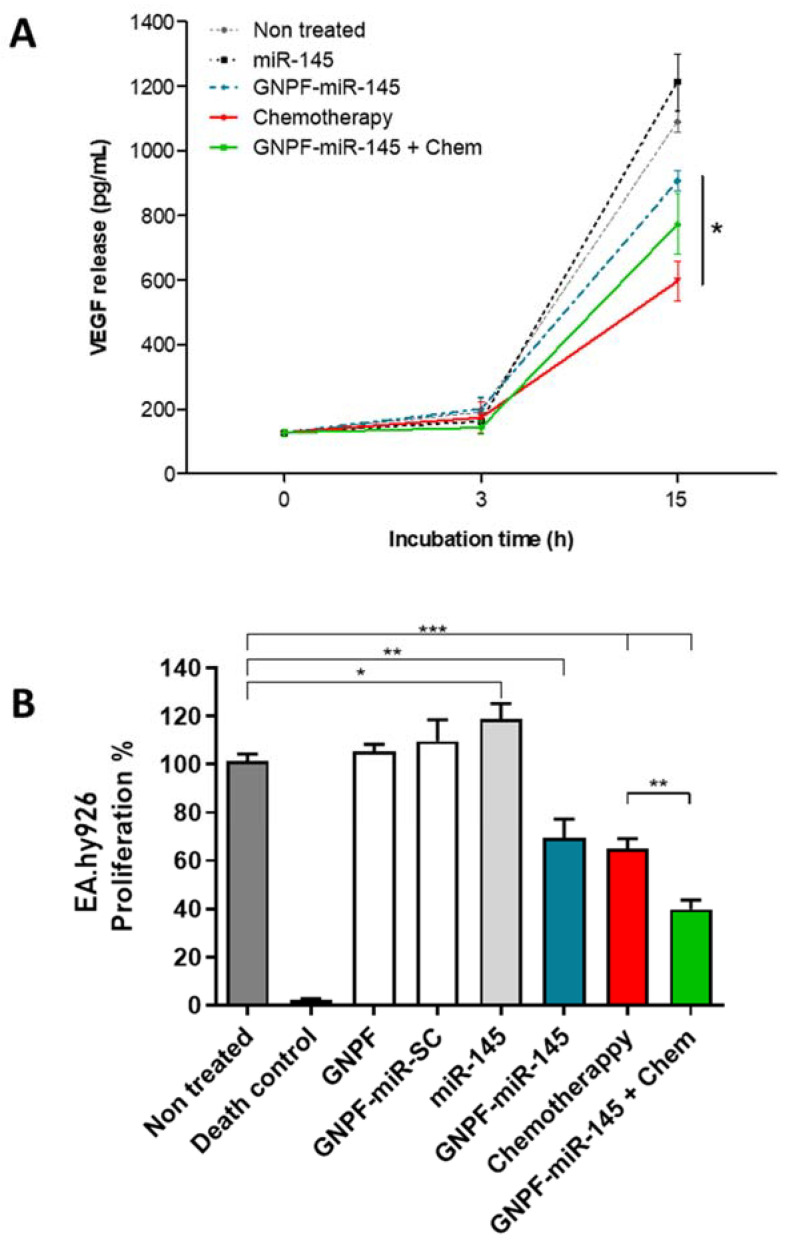
**VEGF release in A2780 cells incubated with GNPF-miR-145 and endothelial cell proliferation.** (**A**) A2780 cells were incubated with GNPF-miR-145, and VEGF medium release was quantified in the culture medium by immunoassay after 0, 3, and 15 h exposure. All data were obtained with *n* = 3 at least in triplicate; error bars: standard deviation; n.s.: not significant; *: *p* <0.05 compared to the control with free miR-145. (**B**) Proliferation assay of EA.hy926 cell line evaluated at 48 h after incubating with the culture medium from A2780 cell line treated with the nanosystems. Data were obtained in triplicate, *n* = 8. For all graphics, error bars represent the standard deviation, * = *p* < 0.05, ** = *p* < 0.01, *** = *p* < 0.001.

To evaluate the effect of GNPF-miR-145 on VEGF release into the medium, an ELISA immunoassay was performed on culture media obtained from A2780 cells incubated with GNPF-miR-145 for 0, 3, and 15 h, and the effect over proliferation of EA.hy926, an endothelial cell line, was evaluated. The results indicated that at 3 h of incubation, no significant differences in the release of VEGF between the treatments were found. However, at 15 h of incubation, the culture media from cells treated with GNPF-miR-145, chemotherapy, and GNPF-miR-145 + chemotherapy showed significantly lower VEGF levels than free miR-145 and controls without treatment. GNPF-miR-145 treatment resulted in 25% less VEGF release compared to free miR-145-treated control. Meanwhile, GNPF-miR-145 + chemotherapy decreased VEGF release by 36%, and chemotherapy by 45% (Figure 6A). This effect was obtained between 3 and 15 h incubation, which correlated with the nanosystem internalization times obtained. 

After 48 h of EA.hy926 incubation with the culture media from A2780 cells of 15 h treated with GNPF-miR-145, the endothelial cells reduced their proliferation by 31%, with chemotherapy by 35%, and GNPF-miR-145 + chemotherapy by 61%, while the medium from free miR-145 treatment, like the control, had no effect, and with VEGF was not diminished (Figure 6B). These results were directly correlated with the quantified levels of VEGF in the media of A2780 cells treated with GNPF-miR-145, resulting in less proliferation of endothelial cells incubated with lower levels of VEGF.

### 3.7. GNPF-miR-145 Effects on Anchor-Independent Growth and Tumoroid Formation

**Figure 7:**
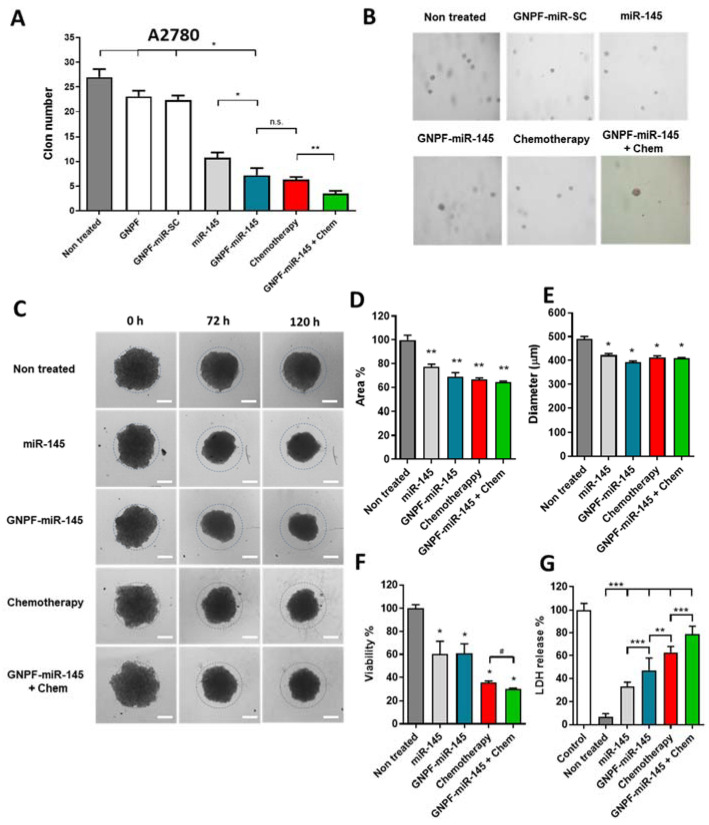
**Effects of GNPF-miR-145 on 3D models.** (**A**) Clone formation in the A2780 cell line against 14 days incubation with a single dose of GNPF, GNPF-SC-miR, miR-145, GNPF-miR-145, chemotherapy, and GNPF-miR-145 + chemotherapy. (**B**) Representative images of clones formed at day 14. The results are the averages of 4 independent assays. For all graphics, error bars represent the standard deviation; * = *p* < 0.05, ** = *p* < 0.01, *** = *p* < 0.001. (**C**) Time-course images captured by optical microscopy of the SKOV3 spheroids after incubation with the nanosystems. Data were obtained in triplicate, *n* = 12; scale bar = 200 µm (**D**) Changes in spheroid area measured at 120 h. (**E**) Changes in spheroid diameter at 120 h. (**F**) Effects on the viability of the spheroids, measured by MTS at 120 h. Data were obtained in triplicate, *n* = 8. (**G**) LDH release assay using the spheroid culture medium after 120 h of the treatments. Data were obtained in triplicate, *n* = 12. For all graphics, error bars represent the standard deviation; # = *p* < 0.05, * = *p* < 0.05, ** = *p* < 0.01, *** = *p* < 0.001.

We next evaluated the ability of ovarian cancer cells to produce progeny under independent anchorage conditions in a soft agar matrix in a three-dimensional (3D) context. After 2 weeks, the number of clones formed from 7000 seeded cells was counted under an optical microscope. We observed that GNPF-miR-145 significantly reduced clone formation, from 27 for the untreated control to 7, showing a reduction of 74%. In this assay, it was evidenced that free miR-145 exerts an effect on clone formation that was not seen in other 2D assays. However, when the miR-145 was carried in the nanoplatform, the inhibition of clone formation was significantly enhanced, resulting in >33% less formation. Of note, the effect of chemotherapy was not different from that exerted by GNPF-miR-145. However, when both therapies were applied together, they had the effect of decreasing the number of clones formed by up to 87%. This indicates that the GNPF-miR145 nanosystem has an important effect, achieving a reduction in anchorage-independent growth, which is considered a correlation parameter of tumorigenicity in vivo. Thus, when acting as a single agent, or in combination with conventional chemotherapy, it is able to decrease tumorigenicity (Figure 7A).

To evaluate the effects of GNPF-miR-145 in a three-dimensional, already formed tumor cell context, individual solid tumor spheroids of SKOV3 line were grown in 96-well plates using the liquid overlay technique (LOT), which consists of the spontaneous grouping of tumor cells on a non-stick surface with a concave bottom to obtain one spheroid per well, which can be evaluated individually as a microtumor [22]. The formed spheroids were exposed to GNPF-miR-145, chemotherapy, and GNPF-miR-145 + chemotherapy treatments. Their morphology, size and area were observed and measured daily under an optical microscope.

On the third day of treatment, significant effects were observed as area reduction (Figure 7D), while on the fifth day of exposure, a considerable decrease in size was evidenced, as shown by a representative image (Figure 7C). The free miR-145 treatment reduced spheroid area by 33%, GNPF-miR-145 by 41%, chemotherapy by 43%, and co-exposure by 46%. All studied conditions showed significant differences compared to the untreated control; however, without significant differences between them (Figure 7D). Likewise, the spheroid diameter decreased from 490 to 392 µm with GNPF-miR-145, which achieved the greatest decrease in diameter, without being significantly different from the rest of the treatments (Figure 7E). Here, we did not observe different effects between free miR-145 and GNPF-miR-145.

### 3.8. GNPF-miR-145 Induces Cell Damage on Formed Tumoroids

To investigate the mechanisms by which GNPF-miR-145 reduces the size of tumor spheroids, at day 5, cell viability, cell damage and apoptosis tests were performed directly on the incubated tumoroids. When evaluating cell viability in intact spheroids, the results showed that both free miR-145 and GNPF-miR-145 decreased viability by 40%, compared to the untreated control. Chemotherapy reduced tumoroid viability by 64%, while GNPF-miR-145 + chemotherapy reduced viability by 70%, maintaining significant differences compared to chemotherapy (Figure 7F). The LDH permeation assay showed that treatment with GNPF-miR-145 caused permeation of 47% with significant differences compared to miR145 (33%), chemotherapy (63%) and GNPF-miR-145 + chemotherapy (79%), compared to the control without treatment (Figure 7G).

In summary, all treatments demonstrated the ability to reduce tumoroid size, causing cellular injury and damage to the plasma membrane. GNPF-miR-145 caused a reduction in viability in a way similar to free miR-145; however, it exerted greater damage to the cell membrane. Interestingly, GNPF-miR-145 + chemotherapy achieved a greater effect, both in terms of reduced viability and plasma membrane damage, compared to chemotherapy. These results are indicative of cell death mechanisms such as necrosis or apoptosis in late stages, explaining the behavior of size reduction in the treated spheroids.

**Figure 8:**
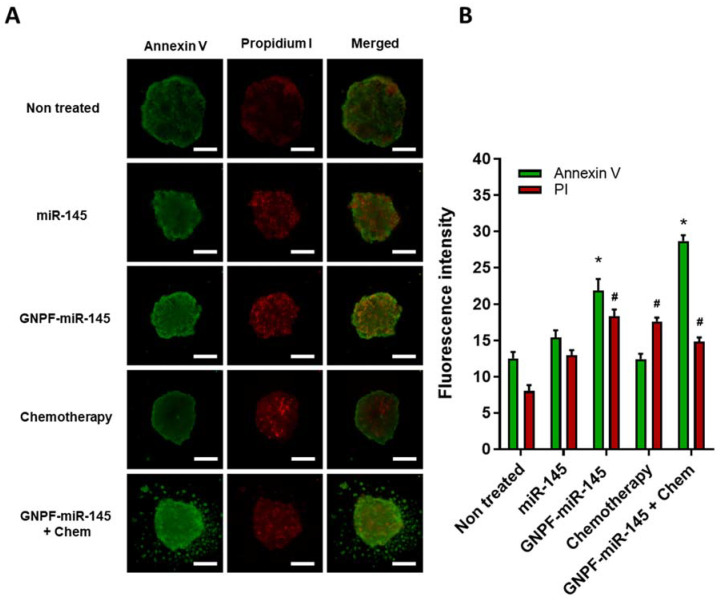
**Annexin V and propidium iodide apoptosis assay in the treated spheroids.** (**A**) Representative fluorescence images of the spheroids after 120 h of treatments, incubated with annexin V and propidium iodide. Scale bar = 200 µm (**B**) Fluorescence quantification of the images. Data were obtained in duplicate, *n* = 3. For all graphics, error bars represent the standard deviation; * = *p* < 0.05 relative to the annexin V untreated control, # = *p* < 0.05 relative to the propidium iodide untreated control.

To understand the underlying mechanisms exerted by the nanosystems, three tumoroids per condition were isolated in a 96-well plate with opaque sides and a light bottom, incubated with annexin V (AV) and propidium iodide (PI), and visualized under a fluorescence microscope. The images show that tumoroids treated with GNPF-miR-145 and GNPF-miR-145 + chemotherapy presented higher fluorescence intensity for AV and IP, revealing positive marks AV (+)/IP (+), which were correlated with late apoptosis or necrosis cell death induced by these treatments. In the case of treatment with conventional chemotherapy, it was observed that the green mark was similar in intensity to the control. However, the red mark was significantly higher (AV (−)/PI (+)), which correlated with necrotic cell death. The results for the miR-145 treatment were not significantly different from the untreated control; in consequence, this treatment did not activate these cell death mechanisms (Figure 8).

## 4. Discussion

In recent years, a significant number of research studies have been directed toward engineering a novel nanosystem that shows good ability to carry therapeutic molecules into tumor cells. In ovarian cancer, nanosystems address the problems of poor solubility and task-specific delivery of chemo-drugs to the targeted site [23].

In this study, we approached this task by engineering a novel nanosystem that includes AuNPs that carry miR-145 and were functionalized with the FSH33 peptide. The nanoplatform was characterized physicochemically, obtaining a final size of 134 nm that was favorable for the arrival of the nanosystem to the cancer cells in a tumor context. It has been described that fenestrations occurring in the tumor vascular endothelium, whose spaces represent sizes from 100 nm–4 µm, allow the passage and retention of nanoparticles in this size range [24]. Despite the wide size range in vascular fenestrations, nanosystems must be able to be internalized into tumor cells. So far, various endocytic cellular mechanisms have been described: clathrin-mediated endocytosis, which internalizes particles smaller than 200 nm, and caveolin-mediated endocytosis, which internalizes endocytic particles larger than 200 nm [25]. Therefore, the nanosystem we designed is optimal to take advantage of the enhanced permeation and retention (EPR) effect in order to reach the tumor interstitium from the blood, as well as to enter the cells through endocytosis, probably via a clathrin-based mechanism.

We also showed that the nanosystem does not affect cellular viability and can specifically interact with ovarian cancer cells due the presence of the FSH33 peptide.

Furthermore the GNPF-miR-145 was able to deliver miR-145 to the cells in a short period of time, elevating levels of gold nanoparticles and miR-145 after 3 h. After 24 h, the levels were diminished; the decrease in gold content could have occurred because the incorporated nanoparticles were being expelled from the cells by excretion mechanisms, such as exocytosis, diffusion, or secretion by extra cellular vesicles [26]. The timing of GNPF-miR-145 incorporation is consistent with the internalization and trafficking of FSH receptor reported in the literature. The described mechanism follows a clathrin-mediated endocytosis model that correlates with the endocytic route and incorporation times achieved for nanoparticles <200 nm [27]. On the other hand, the decrease in miR-145 levels might be due to enzymatic degradation processes, through modifications at the 3′ ends by nucleotide addition or removal [28,29]. Likewise, target-dependent degradation mechanisms have been demonstrated [30], mediated by the strong interaction of miR with its mRNA targets. It has been observed that a high degree of complementarity potently promotes miR degradation through cuts in its structure by the AGO2 miR-processor enzyme [31]. A study carried out by Marzi et al. evidenced that miRs possess different degradation dynamics, with half-life times (T½) of 24 h or even less, obtaining T½ between 4–14 h, which may explain the decrease in miR-145 levels at 24 h in our study [29].

We then studied the toxicity and proliferative effects of the nanosystem, achieving results that agreed with those previously reported, showing that AuNPs obtained through the Turkevich method exert reduced toxicity in vitro, even in small sizes, such as 3.5 nm, or in high concentrations [32,33]. These results suggest that incorporation of nanosystems into cells does not generate alterations in cell viability, or that the mechanisms of toxicity do not yet reflect their effects. This is probably because the molecular systems, both for chemotherapy and those for inhibiting messenger RNA (by miR-145), were still being activated and did not exert a detectable cellular effect at 3 h incubation. In fact, gene knockdown times have been detected as early as 4 h and could last up to even 7 days. For this reason, to demonstrate the functional effects of interferent RNA knockdown as miRs in cell culture, 24–96 h are recommended. Thus, viability effects at shorter times could not be appreciated, but at longer times, when proliferation takes place, they were observed. The proliferation assays indicated that at times greater than 3 h, incubation with GNPF-miR-145 exerted cellular effects, decreasing cell viability in the first instance until 24 h. After replicative cycles occurred, GNPF-miR-145 acted as a proliferation inhibitor, causing permanent cytostatic effects in both cell lines.

The migration of the cancer cells showed a decrease with chemotherapy and with GNPF-miR-145 + chemotherapy. Several authors have reported the effect of miR-145 on cell migration and its relationship with specific molecular targets related to cancer progression [34,35]. Cioce et al. found that the exogenous expression of miR-145 reduces the levels of the OCT4 transcription factor, related to self-renewal of stem cells, which directly regulates the ZEB1 protein gene, related to the epithelium–mesenchymal transition, causing a pro-senescent effect in tumor cells [36]. On the other hand, Hua et al. demonstrated that miR-145 transfection by magnetic nanoparticles directly targets CCND2 oncogenes, involved in cell cycle progression, and E2F3, a transcription factor related to cell proliferation, showing effects on cell migration, invasion, and growth of ovarian cancer cells [37]. The results of migration inhibition are consistent with those reported in the literature, validating the transport of miR-145 by the GNPF [38]. Additionally, VEGF secretion is elevated in ovarian cancer cells, and it was demonstrated that treatment with GNPF-miR145 decreased VEGF secretion to the culture media. When these culture media were used to evaluate the proliferation of endothelial cells (EA.hy926), it was find a decrease in proliferation due to a lack of available VEGF. In line with these results, it has been shown that both VEGF-A and N-RAS are direct targets of miR-145, and that when miR-145 is overexpressed, VEGF levels decrease, causing important inhibitory effects in angiogenesis, both in vitro and in vivo [39]. Furthermore, Garrido et al. found that overexpression of miR-145 decreased VEGF levels in culture media of A2780 and SKOV3 cells together with decreases in other oncogenic proteins. On the other hand, in vivo studies found a decrease in tumor size and metastasis [38].

We also evaluated the ability of ovarian cancer cells in a three-dimensional (3D) matrix, which evidenced that all exposures to the treatments had a direct effect on reducing the tumor size and spheroid area, probably mediated by specific mechanisms of cytotoxicity [40].

Finally, we evaluated whether the nanosystem exerted cell damage on formed tumoroids, and our results correlated with those obtained for 2D cultures, in line with the previously reported evidence. Regarding the mechanisms of cell death, our results were consistent with those found by Wang, who reported that miR-145 can activate the ROCK1/NF-κB apoptotic signaling pathway by inhibiting the expression of ROCK1, which inhibits the cell cycle and thus leads to apoptosis [41].

Previous reports have indicated that miR-145 transfection inhibits tumoroid formation in prostate cancer cells, alluding to the decrease in self-renewal capacity of cancer stem cells, due to inhibition of the CD133 and CD44 markers, along with the Oct4, c-Myc and Klf4 transcription factors related to cell pluripotentiality [42]. On the other hand, Zhu et al. reported that transfection with miR-145 in SW620 colon cancer cells significantly reduced tumoroid formation, proposing the SNAI1 transcription factor as a target, which exerts a critical role in tumor stem cells’ maintenance capacity [43]. Some authors have demonstrated that nanoparticles can enter the tumoroid structure and even reach its nucleus [32]. There are multiple factors that can determine nanoparticle behavior in a 3D context The physicochemical characteristics such as size, surface charge, and molecule coatings are essential to interact with proteins of the medium to create the protein crown or fluid dynamics that promote spheroid entrance [44]. Depending on the nanoparticle configuration, different entry mechanisms can prevail and coexist, such as endocytosis, transcytosis, and transcellular transport, as well as diffusion [45].

So far, few examples of using nanoparticles as miR delivery systems have been described in tumoroids. Wang et al. reported using mesoporous silica nanoparticles to deliver miRs and siRNA in tumor spheroids, achieving penetration and releasing their iRNA cargo through the use of activation by laser irradiation to promote endosomal escape and cell effects [46].

The native three-dimensional structure of carcinomas establishes a number of important interactions for tumor development, such as autocrine and paracrine regulation, oxygen regulation, among others, as well as the differential expression of adhesion, cell contacts, and signaling molecules, which can modify the context of cellular behavior [47,48]. There are also processes that do not occur or that are maybe significantly different compared to what happens in traditional cell culture monolayers. Thus, the encouraging results obtained using these models are often unsuccessful when evaluated in preclinical models. In this way, the tumoroid model appears as an intermediate layer of complexity between two-dimensional cultures and solid tumors in vivo.

## 5. Conclusions

The evaluations carried out on ovarian cancer cells demonstrated the relevance of miR-145 incorporation on a nanosystem structured to achieve better delivery into ovarian cancer cells, taking advantage of the specific presence of FSHR in these types of cells. Thus, the GNPF-miR-145 was effective in transporting the miR inside the cells. In a 2D cell context, the GNPF-miR-145 achieved essential antitumor effects, and the 3D evaluation correlated with this result. Thus, we propose the nanoplatform as a viable system to be used in the next step of preclinical investigation. Several conformational and GNP morphologies have been described: the classic spherical, rod, or star shapes, among others. Nevertheless, the gold nanospheres seem to be the most attractive morphology in terms of obtaining method reproducibility, viable scalability, and manageability, which are relevant points to consider in a candidate for mass production. Therefore, the feasibility of the nanoplatform structure and the simplicity of the miR molecule conjugation present a platform with multiple possibilities for cargo incorporation. Structurally and physiochemically, the miR molecules are almost indistinguishable among them, and the same applies to short RNA molecules such as siRNA. Thus, the nanoplatform could receive a cargo composed of one or multiple short double-strand RNA molecules such as miRs, siRNAs, or mixtures of them, presenting the possibility of creating different, personalized, and powerful tools to achieve a better biological response.

## Data Availability

The data presented in this study are available on request from the corresponding author. The data are not publicly available due to privacy.

## Supplementary Materials

The following supporting information can be downloaded at: https://www.mdpi.com/article/10.3390/pharmaceutics14050958/s1, Figure S1: DLS and AZ graphic of the gold nanosystems; Figure S2: Raman and SERS spectra of nanosystems.

## Abbreviations

AuNP	gold nanoparticles
miR-145	microRNA-145
GNS	gold nanosphere
GNPF	gold nanoplatform
GNPF-miR-145	gold nanoplatform carrying miR-145
EPR	enhanced permeation and retention
GNPF-SC-miR	gold nanoplatform carrying scrambled miR
3D	three-dimensional
LOT	liquid overlay technique
AV	annexin V
PI	propidium iodide
PEG	polyethylene glycol, HS-PEG-NH2
MQ	milli-Q
MES	4-morpholineethanolsulfonic acid buffer
EDC	1-ethyl-3-(3-dymethylaminopropyl
DLS	dynamic light scattering
STEM	scanning transmission electronic microscopy
