# Supplementary material for: Peptide Targeted Gold Nanoplatform Carrying miR-145 Induces Antitumoral Effects in Ovarian Cancer Cells
Source: Pharmaceutics. 2022 Apr 28;14(5):958. doi: 10.3390/pharmaceutics14050958 (PMC9144804; doi:10.3390/pharmaceutics14050958)
Supplement: Supplementary file 1 [file pharmaceutics-14-00958-s001.zip › pharmaceutics-1666363-supplementary.pdf]

pharmaceutics

Supporting Material

Peptide targeted gold nanoplatform carrying miR-145 induces antitumoral effects in ovarian cancer cells

Edison Salas-Huenuleo[1, 2, 3], Andrea Hernández[4], Lorena Lobos-González[2, 5,], Iva Polakovicova[2, 6†], Francisco Morales-Zavala[1, 2, 7], Eyleen Araya[8], Freddy Celis[9], Carmen Romero[2, 4] * and Marcelo J. Kogan[1, 2] *

[1] Laboratory of Nanobiotechnology and Nanotoxicology, Facultad de Ciencias Químicas y Farmacéuticas, Universidad de Chile

[2]Advanced Center for Chronic Diseases (ACCDiS), Universidad de Chile & Pontificia Universidad Católica de Chile

[3] Advanced Integrated Technologies (AINTECH), Chorrillo Uno, Parcela 21, Lampa, Santiago, Chile

[4] Laboratory of Endocrinology and Reproduction Biology, Clinical Hospital, Universidad de Chile

[5] Centro de Medicina Regenerativa, Facultad de Medicina-Clínica Alemana, Universidad Del Desarrollo

[6] Department of Hematology-Oncology, School of Medicine, Pontificia Universidad Católica de Chile, Santiago, Chile

[7] Centro de Nanotecnología Aplicada, Facultad de Ciencias, Universidad Mayor

[8] Departamento de Ciencias Quimicas, Facultad de Ciencias Exactas, Universidad Andres Bello

[9] Laboratorio de Procesos Fotónicos y Electroquímicos, Facultad de Ciencias Naturales y Exactas, Universidad de Playa Ancha, Valparaíso, Chile.

* Correspondence should be addressed to MJK, mkogan@ciq.uchile.cl or CR, cromero@hcuch.cl

1. Dynamic light scattering and Zeta potential distribution profiles

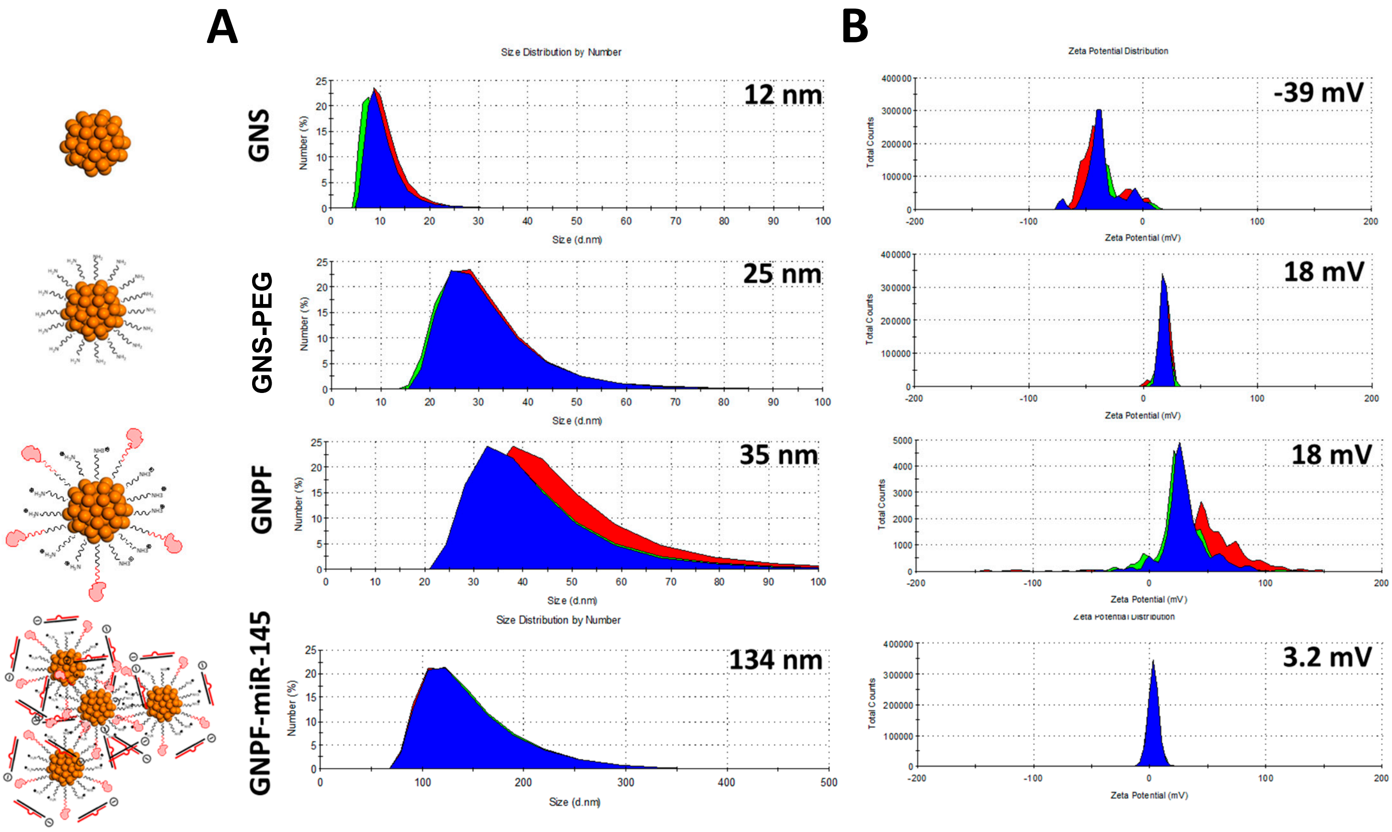

Figure S1. DLS and ZP graphics of the gold nanosystems. (A) DLS graphic of each nanosystem (GNS, GNS-PEG, GNPF and GNPF-miR-145) after its obtention and functionalization. The graphics are the representation of number vs diameter, and are constructed as normal scale (not logaritmic as the graphics from the equipment software). The measurements were maded in triplicate each one with 15 replicates. On each graphic corner the size average appear. (B) Zeta potential graphics of each nanosystem. The graphics are the representaion of total count vs mV. The measurements were maded in triplicate each one with 15 replicates. On each graphic corner the Zeta potential average appear.

2. Raman and SERS spectra of nanosystems

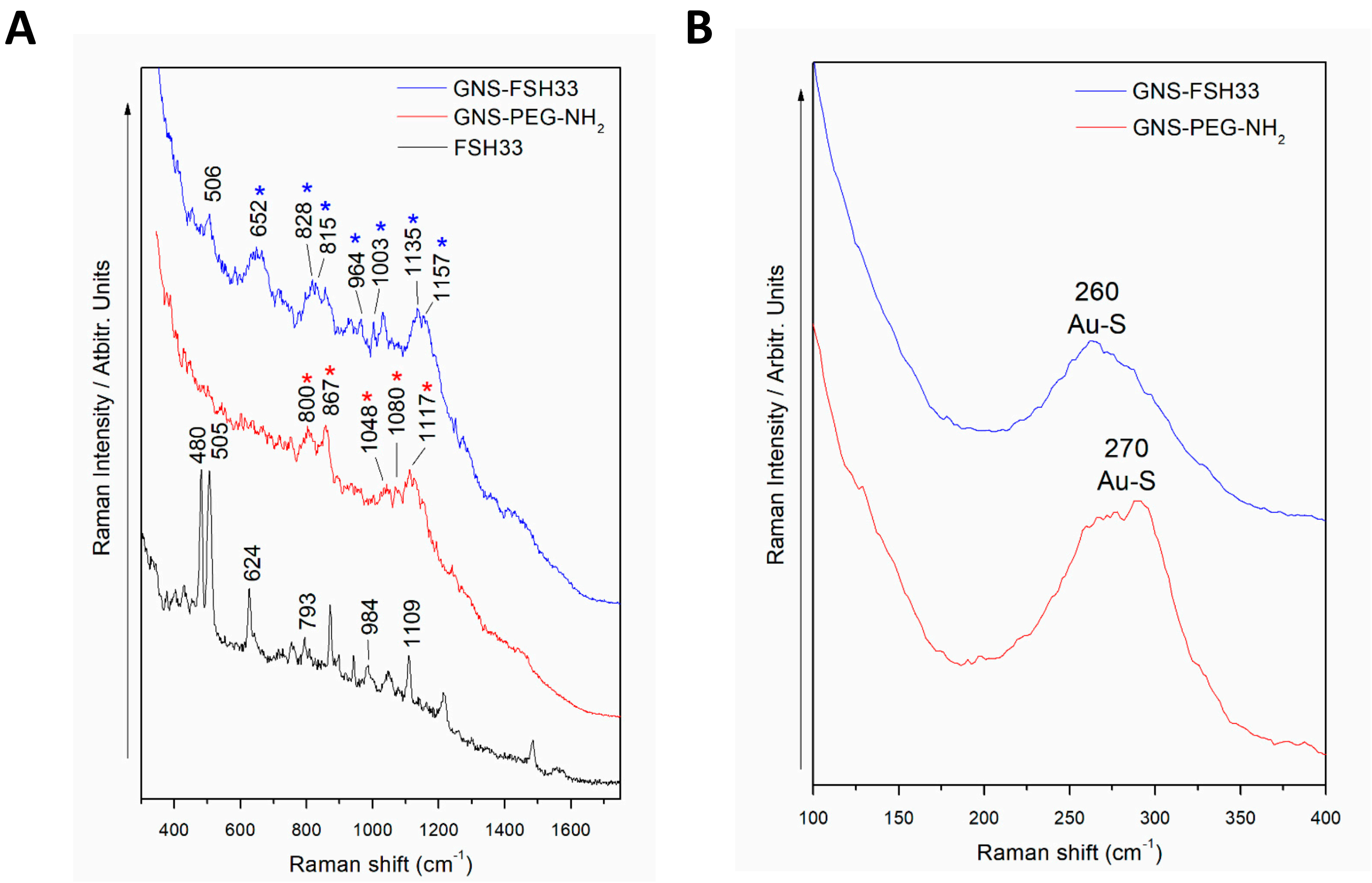

Figure S2. (A) Raman spectrum of FSH33 (black line) and SERS spectra of GNS-PEG (GNS + HS-PEG-NH_2) (red line) and GNPF (GNS + HS-PEG-NH_2 + FSH33) (blue line). All the spectra are displays in the region 300-1750 cm-1. (B) SERS spectra of Au-S vibrational region (100-400 cm-1) of GNS-PEG (GNS + HS-PEG-NH_2) (red line) and GNPF (GNS + HS-PEG-NH_2 + FSH33) (blue line).
